# Natural Environments and Childhood Experiences Promoting Physical Activity, Examining the Mediational Effects of Feelings about Nature and Social Networks

**DOI:** 10.3390/ijerph13040439

**Published:** 2016-04-21

**Authors:** Giovanna Calogiuri

**Affiliations:** Department of Dental Care and Public Health, Faculty of Public Health, Hedmark University College, Hamarveien 112, 2411 Elverum, Norway; giovanna.calogiuri@hihm.no; Tel.: +47-6243-0245

**Keywords:** blue space, exercise, green space, health behaviour change programs, restorative environment, urban green space quality

## Abstract

The importance of natural environments (NEs) for physical activity (PA) has been studied extensively. However, there is scant evidence to explain the motivational processes underlying the NE-PA relation. The aim of this study was to investigate the NE-PA relation using an ecological framework, focusing on perception of NEs, childhood experiences and possible intra- and inter-individual mediators. Data were retrieved from a cross-sectional survey among 2168 adults from all over Norway. In addition, the coverage of NEs by municipalities was retrieved from national registers. Logistic regression showed that, unlike the self-reported proximity to NEs, higher ratings of perceived supportiveness of NEs for PA predicted participation in NE-based PA for at least 60 min/week or 150 min/week, before and after controlling for socio-demographic characteristics. Reporting frequent experiences in nature during childhood was also an important predictor of higher levels of NE-based PA. Furthermore, a mediational analysis showed that the effect of both predictors was mediated by “feelings about nature” and “social networks”. These findings indicate that to encourage the use of local NE for PA, not only should environmental perceptions be taken into account, positive feelings towards nature alongside opportunities to share activity in nature with others should also be promoted.

## 1. Introduction

### 1.1. Physical Activity and Natural Environments

The health benefits of a lifestyle characterized by high levels of physical activity (PA) are well documented. According to the World health Organization (WHO), adults should engage in a minimum of 150 min of moderate-intensity PA a week [1]. Insufficient PA is one of the 10 leading factors in mortality worldwide, with people with insufficient PA levels having 20% to 30% increased risk of death compared to those with sufficient PA levels [2]. In spite of this, it is estimated that, in high-income countries, about 31% of adults are insufficiently physically active [2], and studies using accelerometry suggest that the prevalence of insufficiently active individuals could be even larger [3]. Thus an increase in the levels of PA in the population remains one of the major public health challenges today. According to ecological models, health behaviours such as PA are influenced by multiple levels of factors, including individual, inter-individual and environmental ones, as well as policy. Ecological models have been largely used in PA promotion research and practice [4]. Such models call for *multilevel* interventions, targeting individuals as well as their social and physical environments. In particular it is recommended that environmental interventions ensure safe, attractive and convenient places for PA, alongside motivational and educational programs to encourage their use [4].

Several studies have investigated the relevance of natural environments (NEs) as an arena for weekly PA [5,6], which in the context of promotion of PA could be defined as outdoor green or blue spaces that allow a person to be surrounded by the elements of nature (trees, plants, grass, mountains, water, *etc.*) while engaging in PA [6]. Such environments include urban parks, forests, coasts, *etc.*, which it is claimed are particularly suitable for PA because of their attractiveness and convenience [7]. In fact theories of environmental psychology assign qualities of spontaneous attractiveness to NEs (see, for example, the attention-restoration theory [8] and the biophilia hypothesis [9]), and their restorative effects can lead to positive psychological states such as improved mood and reduced stress [10]. Furthermore, NEs are free for users and can be used for a large variety of activities, including low-threshold PA such as walking, as well as more challenging activities such as running/jogging or participating in outdoor recreation (e.g., hiking, climbing, canoeing). 

The health benefits of PA in NEs go beyond the effects related to just the PA, because in fact exposure to nature can provide additional benefits in terms of mental health and stress reduction [10]. At the same time, the positive emotions experienced when engaging in NE-based PA could have motivational value and support adherence to future PA [6]. Several studies show that people who live near NEs are more likely to meet the recommended levels of PA, but the findings are still mixed [5]. Moreover, there is still scant evidence to explain the motivational processes underlying the NE-PA relation [6]. Studies have investigated participation in outdoor recreation from a philosophical, sociological and educational perspective (see, for example, Gelter [11] and Backman [12]), but its relevance to PA promotion from a public health perspective has still not been explored. In order to develop evidence-based interventions that can encourage and motivate people using available NEs for PA purposes, it is important to understand such processes. 

### 1.2. Natural Environments Promoting Physical Activity: What Are the Determining Factors?

Different studies have focused on self-reported [13,14] or objective [15,16] measurements of the proximity to NEs or their availability close to home, regardless of their characteristics. But the characteristics of an NE (e.g., the type of NE and the features contained in it) seem to predict PA more strongly than mere proximity to *any* NE [17,18,19,20]. The *perceived* environment is also an important influence on people’s PA [4]. There have been discrepancies between objective measurements of the NE and people’s perceptions [21,22]. NEs with a poor field of vision, and with many places where possible danger could be hidden, are less likely to provide a restorative experience, but rather increase stress and negative emotions such as fear [23,24]. This would explain why extensive tree coverage was found to have a negative association with PA [17,19], whereas NEs were more likely to be used when they were perceived as safe and contained features that facilitated PA, such as well-connected trails and playgrounds [17,18]. 

Intra- and extra-individual factors can influence perceptions of an environment as well as the NE-PA relation, e.g., older women were less likely to visit an NE, mainly as a result of barriers such as poor health and poor perceived access to the NE [25]. Furthermore, the way a person values PA and views NEs as a suitable PA arena can influence his or her choice to use available NEs for PA purposes. For example, the environmental quality “compatibility” (the extent to which an environment is compatible with a person’s inclinations at a given moment) predicted the frequency of exercising in NEs [26]. Especially, the extent to which a person was engaged with nature during childhood is an important factor influnecing adult perceptions of and emotional experiences in NEs, and it predicted the frequency of NEs visitations in adult life [27].

The factors influencing the NE-PA relation have been described in the literature [5,6]. However, to date less is known about the *mediators* of the NE-PA relation. Baron and Kenny [28] define a mediator as a third variable that “represents the generative mechanism through which the focal independent variable is able to influence the dependent variable of interest”. In other words, the mediators of the NE-PA relation would explain *how* and *why* the availability of NEs within a person’s living environment leads to enhanced PA. 

From an integrative review of the literature, it emerged that a person’s feelings about nature (e.g., loving nature and/or feeling comfortable and happy in NEs) might be a particularly important intra-individual factor mediating the NE-PA relation [6]. For example, “enjoying nature” was reported to be the most important motive for visiting NEs [29]. A person’s feelings about nature can be boosted by experiences in nature, leading to positive affective states and, possibly, strengthen future PA adherence [6]. Childhood experiences of nature seem to be particularly important in boosting people’s feelings about nature and participation in nature-based activities in adulthood [27,30]. Walking in a pleasant NE was however found to increase participants’ feelings about nature and mediate enhanced affective responses also in adults [31]. 

Inter-individuals factors may also play an important role within the NE-PA relation [6]. Whether a person choses to use NEs as an arena for PA would probably depend on social factors such as companionship and expected social benefits [25,26,32]. “Social interaction” was reported as another important motive for visiting NEs [29], whereas “lack of companionship” was an important reason not to visit them [25]. Some studies also indicate that nearby NEs can foster social interaction, providing opportunities to meet people and strengthening perceived social support and coherence [33,34]. More generally, the extent to which a personis integrated in a network of people who engage in NE-based PA, invite and accompany him or her, is likely to be an important factor influencing the use of available NEs for PA. This could also have a mediational effect in the NE-PA relation, with nearby NEs enhancing people’s social networks and in turn increasing the likelihood that a person would use the NEs for PA. 

### 1.3. The Present Research and Hypotheses

**Figure 1:**
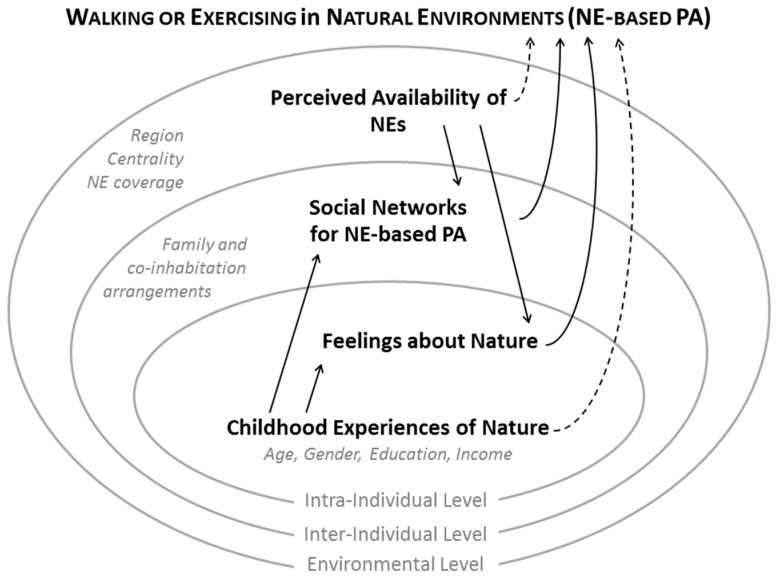
Proposed conceptual model based on an ecological framework that depicts participation in walking or exercising in natural environments (NE-based PA). In bold: predictors and mediators; in italic: control variables. Solid lines: hypothesized *direct* effects; dotted line: hypothesized *indirect* effects.

Knowledge of the determinants of physical activity in natural environments is vital for a PA-promotion perspective, because it would provide better understanding of how to implement motivational and educational programs when encouraging use of NEs for PA. The aim of the present study was to investigate the extent to which perceived availability of NEs and childhood experiences of nature can predict whether or not adult Norwegians participate in NE-based PA in such amounts that can relevantly contribute to meet PA recommendations, and whether intra- and inter-individual factors related to NEs account for the relation. Specifically, the following hypotheses, visually represented in Figure 1, will be tested:

(H1) Participation in NE-based PA for at least 60 or 150 min within a regular week is predicted by the perceived availability of NEs, as indicated by self-reported proximity to NEs and perceived supportiveness of nearby NEs for PA;
(H2) Participation in NE-based PA for at least 60- or 150-min within a regular week is predicted by childhood experiences, independent of other socio-demographic characteristics and the perceived availability of NEs;
(H3) The relationships of participation in NE-based PA with perceived availability of NEs and childhood experiences are mediated by people’s feelings about nature and their social networks for NE-based PA.

## 2. Method

### 2.1. Study Design and Respondents

The dataset for this study was retrieved from a cross-sectional web-based survey initiated by Norsk Friluftsliv in October 2012 [35]; 8620 adults (aged 18+ years) were randomly selected, and stratified by gender, age and geographical area, from a panel of approximately 50,000 individuals who regularly participated in surveys. One reminder was sent to those who had not responded to the first invitation. The final dataset contained data from 2168 respondents (response rate 25%). More details of the sampling and data collection process are available elsewhere [35].

The survey aimed to explore PA habits and motivational factors among Norwegians and included questions on specific PA behaviours, availability of environments close to home and motivational factors for PA. In the present study, only the core variables used to address the study’s purposes are described. All information was self-reported, and all items apart from age and zip code were closed questions with multiple-choice answers. For all questions, alternative response options such as “I don’t know”, “I can’t answer” or “It does not apply to me” were included, to reduce possibly misleading or missing answers. Additional information about the living environment (*i.e.*, NE coverage within the municipality) was derived from registers available from Statistics Norway [36] by cross-matching the respondent’s zip code.

### 2.2. Dependent Variable: Weekly Levels of Participation in NE-Based PA

The respondents were first asked to report “For how long (hours and minutes), in the course of a regular week, do you engage in activities that increase your breathing or make you sweat?” Explanatory examples were provided: “This can include, for example, exercising/training, occupational activities or activities at school, walking/hiking in the forest, and when you walk or bike to and from work or school”. The respondents were then asked to report how much of the time spent on general PA consisted of “walking or exercising in parks, green spaces or natural areas” (*i.e.*, NE-based PA). The resulting continuous variable (minutes per week) was subsequently dichotomized into two dummy variables, defining a lower and a higher level of NE-based PA. The higher PA level was created by setting a cut-off at 150 min/week, chosen because it corresponded broadly to meeting the minimum recommended PA levels in line with the WHO guidelines [1]. It is, however, likely that some individuals do not carry out all their weekly PA in NEs, although they might combine different forms of PA throughout the week. More importantly, NEs might provide an important source of PA for people who do not achieve the minimum recommended levels of PA, but still engage in a substantial amount. Therefore, a lower cut-off point was set at 60 min/week. This cut-off was chosen because figures from national statistics show that a large portion of Norwegians engage in at least 60 min/week [36]; such a pattern was also observed in the study sample, with only 11% engaging in PA for less than 60 min/week. Furthermore, structured exercise sessions are commonly organized in bouts of 30 or 60 min, whereas walking in place of transport or for leisure is commonly organized in bouts of 10–15 min [37]. This cut-off of 60 min/week would therefore include respondents who engaged in organized activities at least once a week, as well as individuals who engage in brief bouts of PA (e.g., walking) on a daily basis. 

Treating amounts of PA as a dummy variable, rather than a continuous variable, has disadvantages due to information loss and consequent reduced statistical variation. However, such an approach is meaningful in a public health perspective, where generally there is a major focus on whether or not people meet PA recommendations or at least engage in substantial amounts of PA that can contribute to achieve the recommendations. A large amount of studies investigating the PA-environment relation have used dummy variables that corresponded to meeting/not meeting PA recommendations, and some of them additionally included a second dummy variable with a lower cut-off point [38,39,40]. Gomez *et al.* [40], for example, used two dummy variables for walking, setting cut-off points at 60 min/week and 150 min/week, as in the present study.

### 2.3. Independent Variables: Measurements of Perceived Availability of NEs and Childhood Experiences

In the present study, the measurements of perceived availability of NEs represented the environmental level of the ecological model. Two measurements of NE were used: *self-reported proximity to NEs* and *perceived supportiveness of NEs for PA*. The former consisted of the self-reported distance (in meters) from the closest NE, irrespective of whether such an NE was perceived as suitable for PA, and was measured through the following item: “How far, more or less, is the closest park, green space or other natural environment from where you live?” Six response alternatives were provided: “less than 100 m”, “between 100 m and 299 m”, “between 300 m and 499 m”, “between 500 m and 1 km”, “more than 1 km” and “I don’t know”. This variable was re-coded ranking the categories from the largest distance (1 = “more than 1 km”) to the smallest distance (5 = “less than 100 m”), excluding the alternative “I don’t know”. 

The perceived supportiveness of NEs for PA was measured from the level of respondents’ agreement with the statement: “From my home, natural environments where I can walk, hike or use for PA are easily accessible”. The extent to which the respondents agreed with such a statement was measured on a scale ranging from 1 to 4 (1 = “it does not suit me”; 4 = “it suits me very well”). An additional rating was “It does not apply to me”, which was not considered for further analysis. This variable is qualitatively different from “self-reported proximity to NEs” as it is mainly a measure of person-environment fit, *i.e.*, the extent to which the respondents perceive NEs as a suitable arena for PA. It has to be noted however that this variable does not measure the respondent’s perceived supportiveness of NEs for PA in general (e.g., relative also to remote or distant NEs), but specifically refers to NEs easily accessible from their home. 

The two measures of perceived availability of NEs were weakly related to each other (ρ = 0.29; *p* < 0.001). Moreover, both measures were also weakly related to objective measurements of NE coverage within the municipality, derived from national registers [36]. The relation was slightly stronger for perceived supportiveness of NEs for PA (ρ = 0.10; *p* < 0.001) than it was for “self-reported proximity to NEs” (ρ = 0.07; *p* = 0.001).

The extent to which the respondents had frequent experiences of nature during childhood was measured as the level of agreement (“How well do the following statements describe your childhood (until you were 16), as far as you can remember it?”) with a set of seven statements, e.g., “I often engaged in walks or hiking trips in the forest” and “I often experienced nature’s quietness”. Each item was rated on a 4-point scale (1 = “it does not suit me”; 4 = “it suits me very well”). A fifth rating point, “I don’t remember,” was also included, but not considered for further analysis. A mean value for these items was calculated and the emerging variable, named “childhood experiences”, was then used for further analysis (Cronbach’s α = 0.75).

### 2.4. Mediators: Inter- and Intra-Individual Aspects of NE-Based PA

The mediators included in this study correspond to the inter- and intra-individual levels of the ecological model. These were measured as the respondents’ level of agreement with a set of statements about their current experiences of nature and NE-based PA. Five statements related to the respondents’ social sphere (e.g., “I know many people who engage in PA in nature” or “I am often invited to participate in some PA in nature”), whereas the other four items related to the respondents’ affective sphere (e.g., “I get in a good mood when I’m in nature” or “I experience positive feelings of mastery when I engage in PA in nature”). The extent to which the respondents agreed with each statement was rated through a scale ranging from 1 to 4 (1 = “it does not suit me”; 4 = “it suits me very well”). An additional item provided the alternative “It does not apply to me”, and was not considered for further analysis. 

Using principal component analysis, a two-component solution was established based on Eigenvalues >1.0, examination of a scree plot and factor loading of ≥0.45. One of the statements (“I enjoy more expeditions in nature when I’m with others”) had factor loading of <0.45, and was therefore removed. No item loaded on both components. Identified components were given descriptive names based on the items showing the larger factor loading, and mean scores were calculated based on the average scoring of the included items. The first component was named “feelings about nature” (Cronbach’s α = 0.86), and relates to the emotional experience of and the value attributed to being in contact with nature. The second component was named “social networks for NE-based PA” (later on referred to in the shorter version “social networks”; Cronbach’s α = 0.74). This variable relates to the type, size and frequency of contacts with the network of people available to the respondent specifically in relation to participation in NE-based PA. Lists of the included items are shown in Table 1.

### 2.5. Covariates

The following covariates were selected after preliminary examination of their individual effect on NE-based PA and collinearity diagnostics:

1. Gender;
2. Age;
3. Educational level (“primary/compulsory education level” up to “university level”; the item “I’m currently studying” was also included);
4. Yearly household income (“≤399,000 NOK”, “400,000–799,000 NOK”, “>800,000 NOK”; the item also included the answer alternatives “I don’t know” and “I don’t want to report it”, which were excluded from further the analysis);
5. Co-inhabitation nucleus, defined as “being married or living with partner”, “living alone”, “living with friends” and “living with parents” (the two latter were pulled together due to small frequency);
6. Whether respondents had responsibility for small children living at home with them permanently or frequently;
7. Region of residence (subsequently grouped into seven major geographical areas);
8. Centrality (living in a “large city”, “small city”, “town/village”, “countryside”);
9. An objective measure of NE coverage within the municipality served as an indicator measure of the *potential* availability of NEs. Information about NE coverage (in square kilometres) for 441 Norwegian municipalities was obtained through a web-based resource of the national statistics agency, Statistics Norway [36], the available data being collected between September 2012 and August 2013 from registries and geographical information systems. The types of NE used for the present study included forests, open spaces of dry land, swampy areas, fresh water, bare mountains, gravel and scree, green spaces and sports fields. The “sports field” coverage is a confounder in this variable, but unfortunately it was impossible to separate it from the measure “green space”. The contribution of these measures, combined with the overall variables, was, however, quite small when compared with the other environments included, so it was deemed that possible confounding effects were, to a certain extent, negligible. The absolute values of NE coverage (km^2^) were subsequently recoded in an interval variable (1 ≤ 100 km^2^; 2 = 100–299 km^2^; 3 = 300–599 km^2^; and 4 ≥ 600 km^2^), to reduce the impact of outliers and obtain better data distribution.

### 2.6. Statistical Analysis

Logistic regression analysis was used to estimate the likelihood of engaging in NE-based PA for ≥60 min/week in relation to each of the independent variables (“self-reported proximity to NEs”, “perceived supportiveness of NEs for PA” and “childhood experiences”), before and after controlling for socio-demographic characteristics. The model was also applied using a cut-off of 150 min/week. All predictors were first tested individually, to estimate their effect on NE-based PA. Subsequently, they were estimated simultaneously, to better establish to what extent they made independent contributions when controlling for the other predictors.

A mediational analysis was then performed to establish the extent of the indirect effect of the perceived availability of NEs and “childhood experiences” on engaging in NE-based PA via “feelings about nature” and “social networks”. According to Baron and Kenny [28], a mediational effect is established if (1) the independent variable is related to the mediator; (2) the mediator is related to the dependent variable and (3) when controlling the mediator, a previously significant association between the independent and dependent variables is no longer significant, or there is a significant reduction in the relation. Therefore, in this study, the mediational analysis followed the following steps:

1. If, a significant effect of the independent variable on NE-based PA was observed, linear regression was used to estimate the relation between the independent variable and the mediators (path *a*).
2. The mediators were added to the model of “self-reported proximity to NEs”, “perceived supportiveness of NEs for PA” and “childhood experiences”, controlled for the socio-demographic characteristics, and the unstandardized coefficients were used to estimate the relation between the mediators and weekly participation in NE-based PA (path *b*). “Feelings about nature” and “social networks” were estimated simultaneously, but also tested individually, to better establish their contribution to the model.
3. The extent of the mediational effect was established using a Monte Carlo method for assessing mediation (MCMAM [41]). According to this method, a mediational effect is considered significant when the confidence intervals for the indirect effects do not include zero (*i.e.*, the null hypotheses of no mediation is rejected). The level of confidence was set as 95%, and the number of repetitions for the simulation was set as 20,000. To make the coefficients for *path a* and *path b* comparable across the equations, a correction was applied using the method described by MacKinnon and Dwyer [42].

The data were initially explored for distribution and outliers, and collinearity diagnostics were run on all variables. Likelihood ratio tests were calculated to compare the logistic regression models’ predictive power. Nagelkerke’s *R*^2^ was used as an estimate of explained variance. Any respondent who selected the alternative response “I don’t know”, “I can’t answer” or “It does not apply to me” for any of the included variables was excluded from the analysis. All analyses were performed using IBM SPSS Statistics version 20.0 (IBM Inc., Chicago, IL, USA); the mediational analysis was performed with the aid of online resources available on N. R. Herr’s [43] and K. J. Preacher’s [41] web pages. The extent of the relation between NE-based PA and the different predictors included in the logistic regression are presented as the odds ratio (OR) and 95% confidence interval (CI). The values of Nagelkerke’s *R*^2^ for explained variance are presented as percentages. Mediational effects are presented as 95% CI. The significance level was set at *p* < 0.05. 

## 3. Results

### 3.1. Sample Characteristics

Information about the sample and the variables used in the study is shown in Table 1. The sample was fairly well balanced with respect to age, gender and geographical distribution, although the groups with lower education, income and PA appear to be somewhat under-represented in relation to the national figures [36]. The values of NE coverage show that most of the respondents lived in fairly “green” municipalities, with about 54% of respondents living in municipalities covered by 50% of the NEs. Most of respondents perceived having good availability of NEs close to home, with 77% of respondents having some NEs within 500 m of home and 55% reporting high levels of perceived supportiveness of NEs for PA. Overall, 51% of respondents reported as engaging in NE-based PA for ≥60 min/week, and 21% engaged in NE-based PA for ≥150 min/week.

### 3.2. Associations of NE-Based PA with Perceived Availability of NEs

The “perceived supportiveness of NEs for PA” predicted participation in NE-based PA before correcting for socio-demographic characteristics, with the likelihood of engaging in NE-based PA for at least 60 min/week or 150 min/week increasing with the growing ratings of the predictor (OR (95% CI) 60 min/week = 1.40 (1.26–1.55); OR (95% CI) 150 min/week = 1.55 (1.34–1.80); *p* < 0.001 for both cut-off points). However, the explained variance was quite small (2% and 3% for the cut-off of 60 min/week and 150 min/week, respectively). “Perceived supportiveness of NEs for PA” was still significant after controlling for the socio-demographic characteristics (OR (95% CI) 60 min/week = 1.39 (1.24–1.56); OR (95% CI) 150 min/week = 1.55 (1.32–1.82); *p* < 0.001 for both cut-off points), with the explained variance increased to 9% and 10% for the lower and higher PA cut-off, respectively. Differently, “self-reported proximity to NEs” did not show any association with participation in NE-based PA, either before (OR (95% CI) 60 min/week = 1.00 (0.94–1.07) 150 min/week = 0.99 (0.91–1.07)) or after controlling for socio-demographic information (OR (95% CI); 60 min/week = 0.99 (0.92–1.06); 150 min/week = 0.97 (0.89–1.05)).

As shown in Table 2, when all the independent variables were added simultaneously in the model, “perceived supportiveness of NEs for PA” remained a highly significant and positive predictor of NE-based PA. Moreover, the inclusion of “self-reported proximity to NEs” in the model did not lead to a noticeably reduction in the effect of “perceived supportiveness of NEs for PA”. On the other hand, “self-reported proximity to NEs” became a significant and *negative* predictor: *i.e.*, the likelihood of engaging in NE-based PA for at least 60 min/week or 150 min/week decreased with increasing proximity to NEs.

### 3.3. Associations of NE-Based PA with Childhood Experiences

A simple logistic regression showed a significant and independent association of “childhood experiences” with participating in NE-based PA for ≥60 min/week (OR (95% CI) 60 min/week = 1.43 (1.25–1.64); *p* < 0.001) and ≥150 min/week (OR (95% CI) 150 min/week = 1.57 (1.33–1.86); *p* < 0.001). The explained variance was, however, somewhat small (2% for both cut-off points). The relation was still significant after controlling for socio-demographic characteristics (OR (95% CI) 60 min/week = 1.37 (1.19–1.58); OR (95% CI) 150 min/week = 1.46 (1.23–1.75); *p* < 0.001 for both cut-off points). The relation remained highly significant also when it was estimated while controlling for “self-reported proximity to NEs” and “perceived supportiveness of NEs for PA” (Table 2). Notably, the inclusion of “childhood experiences” in the model did not lead to much reduction in the effect of “perceived supportiveness of NEs for PA”, nor did the inclusion of “perceived supportiveness of NEs for PA” and “self-reported proximity to NEs” lead to a reduction of the effect of “childhood experiences”. 

Among the respondents’ socio-demographic characteristics, age was a significant predictor of engaging in NE-based PA for both, ≥60 min/week and ≥150 min/week, with the likelihood increasing with growing age (Table 2). Region of residence was also a significant, though weaker, predictor of engaging in NE-based PA, with the respondents who live in Western Norway having an increased likelihood of engaging in NE-based PA for ≥150 min/week (Table 2). There were no noteworthy changes of the socio-demographic characteristics when the predictors were tested individually or when they were estimated simultaneously.

### 3.4. Mediational Analysis

Since an independent association of “self-reported proximity to NEs” with NE-based PA was not found, the mediational analysis was performed only to examine possible indirect effects of “perceived supportiveness of NEs for PA” and “childhood experiences” on participation in NE-based PA for at least 60 min/week or 150 min/week. A linear regression with “perceived supportiveness of NEs for PA” as independent variable showed a significant and independent positive association with both “feelings about nature” (β = 0.40; *t* = 20.16; *p* < 0.001) and “social networks” (β = 0.34; *t* = 16.57; *p* < 0.001). Linear regression showed also that “childhood experiences” was positively related to both “feelings about nature” (β = 0.41; *t* = 20.81; *p* < 0.001) and “social networks” (β = 0.34; *t* = 16.74; *p* < 0.001). These findings confirm that these variables qualify as possible mediators of the NE-PA relation. 

When added to the logistic regression, both, “feelings about nature” and ”social networks” predicted participation in NE-based PA at both cut-off points, with the likelihood of engaging in NE-based PA for at least 60 min/week or 150 min/week increasing with increasing ratings of the mediators (Table 3). It should also be noted that when these two variables were simultaneously included in the model, the explained variance was almost doubled compared with the model shown in Table 2, increasing it by 8% when applying the 60 min/week cut-off and 7% when applying the cut-off of 150 min/week. When the mediators were applied individually, the increase in pseudo R^2^ was 5%–6% for “feelings about nature”, and 5%–4% for “social networks”, respectively for the 60 min/week and 150 min/week cut-off. The contribution of the mediators to the model varied, however, for the different cut-off: when applying 60 min/week as the cut-off, “social networks” was a stronger predictor than “feelings about nature”, whereas it was reversed when the model was applied to the cut-off of 150 min/week. In the model applied to the cut-off of 60 min/week, the OR for “social networks” was also larger than the model applied to the cut-off of 150 min/week, whereas again it was reversed for “feelings about nature” (Table 3). 

When the mediators were added to the model, the relation between “perceived supportiveness of NEs for PA” and NE-based PA was substantially reduced, to the point that it was no longer significant (see Table 2 *vs.* Table 3). When the mediators were tested individually, the inclusion of “feelings about nature” completely reduced the relation of “perceived supportiveness of NEs for PA” with NE-based PA at both cut-off points, whereas the inclusion of “social networks” only led to partial reductions (OR (95% CI) 60 min/week = 1.16 (1.02–1.32); OR (95% CI) 150 min/week = 1.33 (1.11–1.59); *p* = 0.023 and 0.002, respectively). The MCMAM testing the indirect effect of “perceived supportiveness of NEs for PA” and participation in NE-based PA confirmed statistical significance of the mediational effect at both levels of NE-based PA, as shown by the confidence intervals for the indirect effects that did not include zero in any of the cases: the 95% CI for the indirect effect mediated by “feelings about nature” were 0.02 to 0.04 and 0.03 to 0.06 for the 60 min/week and the 150 min/week cut-off, respectively; the 95% CI for the indirect effect mediated by “social networks” were 0.02 to 0.04 and 0.01 and 0.03 for the 60 min/week and the 150 min/week cut-off, respectively. 

The relation between “childhood experiences” and participation in NE-based PA at both cut-off points was also completely reduced when they were estimated simultaneously with “feelings about nature” and “social networks” (Table 3). Moreover, the association of “childhood experiences” with NE-based PA was completely reduced also when the mediators where tested individually. A mediational effect was confirmed by the MCMAM at both PA cut-off, as shown by the confidence intervals for the indirect effects that did not include zero in any of the cases. The 95% CI for the indirect effect mediated by “feelings about nature” were 0.02 to 0.04 and 0.03 to 0.06 for the 60 min/week and the 150-min/week cut-off, respectively; the 95% CI for the indirect effect mediated by “social networks” were 0.02 to 0.04 and 0.01 to 0.04 for the 60-min/week and the 150 min/week cut-off, respectively. 

## 4. Discussion

### 4.1. Summary of Results

The purpose of this study was to investigate the extent to which perceived availability of NEs (*i.e.*, the perceived supportiveness of NEs for PA and proximity to NEs) and childhood experiences of nature predict participation in NE-based PA in Norwegian adults, and whether intra- and inter-individual factors (*i.e.*, feelings about nature and social networks for NE-based PA) account for these relations. The findings show that, unlike the self-reported proximity, the perceived supportiveness of NEs for PA increased the likelihood of participating in NE-based PA. The relation was significant before and after controlling for socio-demographic characteristics. Further, significant associations were found for both a lower (60 min/week) and a higher (150 min/week) cut-off of weekly participation in NE-based PA. More frequent childhood experiences in NEs also increased the likelihood of engaging in lower (60 min/week) and higher (150 min/week) levels of NE-based PA. Moreover, when childhood experiences and perceived supportiveness of NEs for PA were simultaneously tested in a multiple logistic regression, their effect was not much reduced, indicating that both predictors make independent contributions in predicting high levels of NE-based PA. As hypothesized, both the effects of perceived supportiveness of NEs for PA and childhood experiences of nature were significantly mediated by feelings about nature and social networks.

Some of the individual socio-demographic characteristics included in this study influenced the NE-based PA. Among these, age was the most consistent across the models, and in fact was a significant positive predictor, with the likelihood of engaging in NE-based PA for at least 60 min/week or at least 150 min/week increasing with age. Previous studies found mixed results regarding the relation between age and use of NEs for PA [5,6]. However, this finding indicates that, in Norway, NEs are an important source of PA especially for older individuals, and this has a particular relevance in public health, given the fact that general PA levels tend to decrease with growing age. Other associations were found for region of residence, with people living in Western Norway having an increased likelihood of engaging in higher levels of NE-based PA (*i.e.*, ≥150 min/week). Preliminary analysis found however that this association could be partly explained by objective environmental characteristics, as the effect of region was reduced if NE coverage within the municipality was controlled for. Gender was also associated with higher levels of NE-based PA, with men being more likely to engage in NE-based PA for 150 min/week or more compared with women, although such an association emerged only after including feelings about nature and social networks in the model.

### 4.2. Physical Activity is Predicted by Perceived Supportiveness of NEs for PA to a Greater Extent as Compared with Self-Reported Proximity to NEs

The findings of this study indicate that perceiving nearby NEs to be highly supportive for PA, not simply the proximity to *any* NE, can help people meet the minimum recommended levels for weekly PA. Noticeably, the proximity to NE, which was not associated with NE-based PA when tested individually, became a *negative* predictor of NE-based PA when the perceived supportiveness of NEs for PA was controlled for. Therefore, the negative association between proximity to NEs and NE-based PA ought to be considered in relation to the perceived supportiveness of the proximate NEs for PA: when one has availability of NEs nearby home, but these NEs are not perceived as a valuable arena for PA, one is less likely to use them for PA purposes.

This finding confirms the importance of environmental perceptions, especially with respect to the perceived supportiveness for PA, in determining whether or not people use NEs for PA purposes. Different studies showed that proximate NEs are more likely to be used when they are attractive and have features that facilitate PA, such as high aesthetic/restorative value [44,45] and the presence of a playground and well-connected tracks/trails [17,18]. However, to some extent, the findings of the present study conflict with those of some previous studies. Proximity to NEs was, in fact, reported to predict PA [13,19,39,46,47], but the findings of these studies are somewhat mixed. First, not all proximate NEs were associated with PA. Shores *et al.* [46] and Mowen *et al.* [47] found that having urban parks near home was associated with increased PA, although Foster *et al.* [39] found that the relation was significant only in men. On the other hand, Wilson *et al.* [19] found no association between PA and having a park near home of home, whereas one was found for proximity to coasts/rivers. Moreover, these studies measured overall PA, such as walking [19,39], or whether the respondents met the minimum recommended PA levels [46,47]. Therefore, it was not clear to what extent the increased activity levels were actually related to the use of NEs. Lackey and Kaczynski [21] found, for example, that having a park near home was not associated with park-based PA, whereas park-based PA was more likely when people were aware of having a park close to their home. In Lackey and Kaczynski’s study it was also found that some characteristics of the parks, such as containing a playground or a wooded area, increased the likelihood of people being aware of local parks. 

However, it should not be concluded simply that proximity to NEs is unimportant. The health potential of NEs goes beyond regular participation in PA. NEs can provide restorative experiences, helping people to cope with stress [14,48], and health benefits even if people visit them occasionally [49]. Moreover, attractive natural sights can encourage other forms of PA, such as walking [45] or jogging [50] in the neighbourhood. In Norway people in general have quite good access to NEs such as forests, coasts, and open spaces, even in the immediacies of urban areas. This is reflected in the sample, as in fact overall 77% reported having an NE within 500 m of home and only 8% did not have a NE within 1 km of home. This is also likely to explain partly why an individual significant relation between proximity to NEs and NE-based PA was not found, and why the relation became negative when perceived supportiveness was controlled for. Nevertheless, this finding targets the importance of the quality of NEs, and the way people perceive them, in promoting NE-based PA.

### 4.3. Perceived Supportiveness of NEs for PA and Childhood Experiences Make Independent Contributions in Predicting High Physical Activity Levels

The importance of childhood experiences of nature has been advocated, not only for its possible impact on PA in adult life, but also because of its important role in promoting a healthy connection with the natural world, as well as its relation to good mental and physical health [51,52]. The present study confirms that people who had more frequent experiences in nature during childhood are more likely to engage in higher levels of NE-based PA in adult life. This finding is in line with what was previously found by Ward Thompson *et al.* in an English and a Scottish sample, which showed that the frequency of experiences in nature during childhood was a highly significant predictor of how often people visit NEs in adulthood, and it was overall the most effective predictor among other socio-demographic variables [27]. However, the study by Ward Thompson *et al*. also found that the “childhood factor” influenced adult perceptions of and emotional experiences in NEs [27]. This implies that frequent childhood experiences in NEs might influence adult participation in NE-based PA not only by strengthening their emotional attachment to nature and NE-related networks, but also by influencing the perceived supportiveness of NEs for PA. However, in the present study, it was found that when perceived supportiveness of NEs for PA and childhood experiences where simultaneously included in a multiple logistic regression, they both remained highly significant, without much reduction in the effect on NE-based PA. This finding suggests that both predictors make largely independent contributions in predicting high levels of NE-based PA. Thus perceptions of NEs are not ultimately determined by childhood experiences; other factors, such as the actual physical characteristics of the NE, also play an important role.

### 4.4. The Relations of Physical Activity with Perceived Supportiveness of NEs for PA and Childhood Experiences are Mediated by Feelings about Nature and Social Networks

A large body of literature has investigated the environmental correlates of PA [4], also in relation to NEs [5]. The present study, however, provides further evidence of the mechanisms underlying the NE-PA relation, which to date are not fully documented [6]. Such knowledge is important for implementing evidence-based interventions for motivating people who use NEs for PA. It is known that pleasant experiences of nature can lead to increased feelings of connectedness with nature [31] and the pleasure or desire of being in contact with nature can motivate people to visit NEs [29]. Interestingly, this study indicated that having more positive feelings about nature is more important for those who engage in larger amounts of NE-based PA (*i.e.*, ≥150 min/week), whereas it was less important for those who engage in NE-based PA for at least 60 min/week. This is somewhat in line with perspectives described by Gelter, who argues that strong emotional responses to being in contact with nature can lead to an emotional attachment to nature, which can sustain participation in activities that allow the individual to be in close relation with the NE [11]. 

The importance of social networks as a predictor of nature-based PA is also consistent with previous research. It has been previously shown that visiting and spending time in a NE are often perceived as an opportunity to interact with others [14,29]. This can enhance and strengten networks of people in the context of NE-based activities. Within such networks, members can provide mutual support such as accompany or invite each other. For example, social aspects such as companionship, expected social benefits and social cohesion, have in fact consistently been found to influence PA behaviours in general, as well, whether or not people use NEs for PA purposes [25,26,32]. In this study, “being often invited to participate in NE-based PA” had the strongest factor load, suggesting that this aspect is particularly important. It emerges, however, that the support provided by such networks might be more important for those who engage in smaller amounts of NE-based PA (*i.e.*, ≥60 min/week), whereas those who engage in NE-based PA for 150 min/week or more appear to rely on their networks to a lesser extent. This is also in line with previous studies showing that people who rely more on social support as a motivational strategy to engage in regular walking, tend to walk less frequently than people who give less importance to social support [53]. 

As discussed above, childhood experiences of nature was unable to ultimately account for the NE-PA relation. This finding suggests that nature experiences in adult life, promoted by proximate quality NEs, can also foster feelings for nature and social networks, which will in turn help sustaining high levels of weekly NE-based PA. Previous studies showed that it is not only childhood experiences of nature that have a positive impact on people’s feelings of connectedness to nature. For example, Mayer *et al.* [31] also showed increases in connectedness to nature after brief walks in NEs in a sample of young adults. Moreover, exposure to nature while engaging in PA has been consistently shown to provide positive emotional responses, such as improved mood, and to reduce stress [6,10]; this, in turn, might positively impact on people’s feelings about nature and their perception of NEs as an arena for PA and recreations. This implies that even though a person was not fortunate enough to have frequent experiences of nature during childhood, emotional connections with nature might be nourished in adult life through positive experiences of nature.

### 4.5. Strength and Limitations of the Study

To the best of the author’s knowledge, this is the first study investigating motivational processes underlying the NE-PA relation in a large population. The inclusion of affective perceptions of the NE (*i.e.*, feelings about nature) is also a novelty in the field, and provides perspectives that might more closely relate to the motivational process for future participation in PA. Moreover, concepts like connectedness with nature or attachment to green space have been studied in relation to health [54] and pro-environmental behaviour [55], but not yet in relation to PA.

There are, however, a number of limitations and weaknesses to acknowledge. The sample was well balanced for gender, age and geographical distribution, but the respondents’ socioeconomic status was higher when compared with national statistics [36]. Another limitation concerns the self-reported measurement of PA, which could have overestimated effect measures. Furthermore, moderate- and vigorous-intensity PA were conflated. Similar instruments have previously been used by Norwegian national institutions [36], as well as in scientific studies [33,56], but this instrument is nevertheless likely to underestimate PA levels for individuals who would otherwise meet WHO recommendations for *vigorous*-intensity PA. The dataset lacked important information that is likely to have an impact on the model, e.g., ethnicity, health status, occupational status and knowledge/experience of relevant policies. The overall explained variance of the proposed model is quite small (17%), but such a level of explained variance is not far from previous application of different social–cognition models [57]. Treating amounts of PA as a dummy variable, rather than a continuous variable, might also have limitations. However, such an approach is meaningful in a public health perspective, where there is interest in understanding to what extent NE can help people meeting PA recommendations, or at least encourage some substantial amount of PA. The fact that this was a cross-sectional study does not allow confirmation of the causation of the relation. Especially, it was suggested that the NE-PA relation could be biased by self-selection, with people who are more active choosing to live where environments that support PA are available. though some studies seem to reject such a possibility [58,59]. However, only longitudinal prospective studies could really address this issue. Last but not least, as a result of its small population density and large areas of NEs in Norway, the NE-PA relation is likely to differ from that of other countries, and their generalization to other countries is limited. Replication in different environmental/cultural settings is recommended.

### 4.6. Implications

Apart from the environmental factors, ecological models typically include a level related to policies, which was unfortunately not measured in this study. To better contextualize the findings and their possible implications, a brief overview of the policies and organizations related to NE-based PA in Norway is provided. In Norway, as a general policy regulating participation in NE-based PA, the right to roam (*allemannsrett*, literally “every man’s right”) prevails; this is the right to access public and, to a certain extent, privately owned land for recreation and exercise [60]. Another important policy is the fact that *friluftsliv*, the Scandinavian concept of outdoor life and recreation, is formally included in the physical education curriculum in compulsory schooling [61]. At an organizational level, in Norway there is a network of *friluftsliv* organizations, with over 760,000 members and more than 4600 clubs and associations, which have, as part of their scope, experience of nature and outdoor activities. There is also an umbrella organization (Norsk Friluftsliv), the purpose of which is to promote traditional and nature-friendly outdoor recreations and the right to roam. Altogether, the environment policy related to outdoor recreations, alongside the extensive NE coverage and the lively “*friluftsliv* culture”, could make Norway or other countries with similar characteristics a particularly favourable setting for implementing PA and health-promotion interventions that focus on NEs.

According to an ecological perspective, to effectively impact PA levels in the community environmental interventions should be integrated with motivational programs that act at individual and inter-individual levels [4]. In particular, based on the findings from the present study and the state of the art in the field, it is recommended that multilevel interventions do the following:

1. Protect and enhance access to NEs that support PA and are well suited to the needs and profile of local communities. It should be noted that, as opposed to other studies available in the literature, the variable used in this study did not specify what type of NE, or feature contained within it, was perceived as “supportive for PA”. It is expected that individuals from different backgrounds and with different preferences for PA assign different values to NEs, to the extent to which they consider them suitable for PA [62]. For example, some people with more experience of NE-based PA are likely to be more comfortable and perceive fewer barriers in NEs such as forests or open mountains, whereas other less experienced individuals might see these NEs as almost inaccessible. It is therefore especially important to take into account the way local communities perceive the available NEs; therefore “bottom-up” approaches for improvements of the local NEs should be considered.
2. Foster supportive social networks for NE-based PA, e.g., facilitating *buddy systems*, programming walking/exercising groups in local NEs and/or organizing internet communities. This approach should target particularly those who engage in NE-based PA in small or irregular amounts.
3. Elicit enhanced feelings about nature by promoting positive experiences in pleasant NEs and activities that aim to re-connect people with the natural world. Such interventions should target children especially, but can and should also involve adults. More attention should be put into encouraging NE-based PA in women as they were found to be less likely to engage in higher amounts of NE-based PA.

## 5. Conclusions

The findings of this study show that the extent to which natural environments promote and help sustain higher amounts of physical activity hardly depends on simply having *any* natural environments near home. On the other hand, the extent to which an individual perceives this environment to be supportive for physical activity has an important impact. Such an effect is mediated by increased emotional attachment to nature and having a network of people who can accompany or invite them. Moreover, more frequent childhood experiences of nature can positively influence the relation by strengthening people’s feelings about nature and their perceived social networks for NE-based PA. Such findings have implications for planning and implementing interventions to encourage greater amounts of physical activity in the community, emphasizing the importance of taking into account individual perceptions of local natural environments, as well as the importance of integrating environmental interventions with programs that act at a social and an individual level. 

## Figures and Tables

**Table 1:** Background information and physical activity levels in a sample of 2168 Norwegian adults.

Variable	Total Sample (*n* = 2168)
**Gender (%)**	
Male	50.4
Female	49.6
**Age (%)**	
18–44 years	30.3
45–64 years	48.7
>65 years	21.0
**Education (%)**	
≤10 years (compulsory)	8.4
11–13 years (high school)	28.1
>13 years (higher education)	46.1
Currently studying	17.4
**Household income (6 NOK ≈ 1 US$) (%)**	
≤399,000 NOK	15.7
400,000–799,000 NOK	40.8
>800,000 NOK	33.7
Missing	9.8
**Co-inhabitation nucleus (living with …) (%)**	
Parents or friends	4.6
Alone	26.1
Spouse or partner	69.3
**Small children at home (%)**	
Living with children	28.9
No children	71.1
**Region (major geographical areas) (%)**	
Oslo and Akershus	25.9
Hedmark and Oppland	8.1
South-eastern Norway	18.5
Agder and Rogaland	11.7
Western Norway	17.8
Trøndelag	8.4
Northern Norway	9.7
**Centrality (%)**	
Large city	36.0
Small city	24.1
Town/village	24.2
Countryside	15.7
**NE coverage within the municipality (absolute values) (%)**	
<100 km^2^	17.3
100–299 km^2^	28.1
300–599 km^2^	33.0
≥600 km^2^	20.4
Not available	1.1
**NE coverage within the municipality (adjusted for the municipality’s size) (%)**	
≤25%	10.4
26%–50%	25.1
51%–75%	29.2
76%–100%	24.9
Not available	1.1
**Self-reported proximity to NEs (%)** “*How far, more or less, is the closest park, green space or other natural environment from where you live?*”	
<100 m	41.2
100–199 m	23.1
200–499 m	12.6
500 m to 1 km	15.2
>1 km	6.3
I don’t know	1.6
**Perceived supportiveness of NEs for PA (%)** “*From my home, natural environments where I can walk, hike or use for PA are easily accessible*”	
It suits me very well	55.4
It suits me rather well	29.0
It suits me little	15.1
It does not suit me	2.9
Not applicable	0.4
**Walking or exercising in natural environments (NE-based PA) (%)**	
<60 min/week	49.9
60–149 min/week	30.4
150+ min/week	20.5
**Other forms of leisure-time PA (%)** *(organized sports, exercise in the gym or other activities)*	
<60 min/week	60.9
60–149 min/week	21.3
150+ min/week	17.8
Childhood experiences (Mean ± SD) * Included items:	2.34 ± 0.63
*I often experienced nature’s quietness*	
*I often engaged in walks or hiking trips in the forest*	
*I often engaged in walks or hiking trips in the mountains*	
*I often visited the seaside*	
*I often went fishing*	
*I often went skiing*	
*I often engaged in orienteering*	
Feelings about nature (Mean ± SD) * Included items (ranked by factor loading):	3.14 ± 0.72
*I get in a good mood when I’m in nature*	
*I like nature’s quietness*	
*Experiences of nature are important to me*	
*I experience positive feelings of mastery when I engage in PA in nature*	
Social networks for NE-based PA (Mean ± SD) * Included items (ranked by factor loading):	2.26 ± 0.67
*I am often invited to participate in some PA in nature*	
*I often engage in NE-based PA together with friends*	
*I often engage in NE-based PA together with my children*	
*I know many people who engage in PA in nature*	

Note: * Ratings of agreement 1 = “it does not suit me”; 4 = “it suits me very well”.

**Table 2:** Logistic regression of self-reported proximity to NEs, perceived supportiveness of natural environment for physical activity and childhood experiences predicting different levels of physical activity in Norwegian adults, controlling for socio-demographic characteristics.

Predictors	Walking or Exercising in NEs (NE-Based PA)					
	Cut Off 60 min/Week (*n* = 2137)			Cut Off 150 min/Week (*n* = 2137)		
	*p*	OR	95% CI	*p*	OR	95% CI
Self-reported proximity to NEs	0.039	0.93	(0.86–1.00)	0.013	0.89	(0.82–0.98)
Perceived supportiveness of NEs for PA	<0.001	1.38	(1.22–1.56)	<0.001	1.57	(1.32–1.86)
Childhood experiences	0.001	1.30	(1.12–1.50)	0.001	1.36	(1.14–1.63)
Gender						
Male	-			-		
Female	n.s.	1.11	(0.93–1.33)	n.s.	0.89	(0.71–1.11)
Age (years)	<0.001	1.03	(1.02–1.04)	<0.001	1.03	(1.02–1.04)
Education level (years)						
≤10 (compulsory)	-			-		
11–13 (high school)	n.s.	1.06	(0.74–1.52)	n.s.	0.88	(0.58–1.35)
>13 (higher education)	n.s.	1.15	(0.81–1.62)	n.s.	0.92	(0.62–1.37)
Currently studying	n.s.	1.09	(0.74–1.61)	n.s.	0.82	(0.52–1.30)
Income (NOK) (6 NOK ≈ 1 US$)						
≤399,000	-			-		
400,000–799,000	n.s.	0.88	(0.68–1.13)	n.s.	0.82	(0.59–1.12)
>800,000	n.s.	1.02	(0.77–1.35)	n.s.	1.11	(0.78–1.56)
Missing	n.s.	0.97	(0.68–1.38)	n.s.	0.86	(0.55–1.35)
Co-inhabitation nucleus						
Living with parents/friends	-			-		
Living alone	n.s.	1.12	(0.66–1.91)	n.s.	0.83	(0.39–1.77)
Living with spouse/partner	n.s.	1.36	(0.81–2.27)	n.s.	1.06	(0.51–2.21)
Having small children						
No	-			-		
Yes	n.s.	1.07	(0.86–1.32)	n.s.	0.89	(0.68–1.18)
Region						
Oslo and Akershus	-			-		
Hedmark and Oppland	n.s.	0.81	(0.55–1.19)	n.s.	0.78	(0.47–1.29)
South-eastern Norway	n.s.	0.89	(0.66–1.21)	n.s.	0.92	(0.63–1.36)
Agder and Rogaland	n.s.	1.34	(0.98–1.83)	n.s.	1.20	(0.82–1.76)
Western Norway	n.s.	1.24	(0.94–1.62)	0.019	1.49	(1.09–2.07)
Trøndelag	n.s.	0.95	(0.67–1.34)	n.s.	1.26	(0.82–1.93)
Northern Norway	n.s.	0.79	(0.54–1.15)	n.s.	1.02	(0.64–1.63)
Centrality						
Large city	-			-		
Small city	n.s.	0.91	(0.70–1.18)	n.s.	1.23	(0.90–1.69)
Small town	n.s.	0.89	(0.69–1.14)	n.s.	1.01	(0.74–1.38)
Countryside	n.s.	0.92	(0.68–1.23)	n.s.	1.31	(0.92–1.66)
Municipality’s NE coverage (km^2^)	n.s.	1.04	(0.94–1.16)	n.s.	0.99	(0.87–1.12)
**Explained variance (Nagelkerke’s *R*^2^) (%)**	**10**			**11**		

Note: n.s. = non-significant.

**Table 3:** Logistic regression of self-reported proximity to NEs, perceived supportiveness of natural environment for physical activity and childhood experiences of nature predicting different levels of physical activity in Norwegian adults, after controlling for socio-demographic characteristics and possible mediators.

Predictor	Walking or Exercising in NEs (NE-Based PA)					
	Cut-Off 60 min/Week (*n* = 2134)			Cut-Off 150 min/Week (*n* = 2134)		
	*p*	OR	95% CI	*p*	OR	95% CI
Feelings about nature	<0.001	1.68	(1.42–1.98)	<0.001	2.04	(1.62–2.56)
Social networks for NE-based PA	<0.001	1.88	(1.59–2.23)	<0.001	1.67	(1.37–2.05)
Self-reported proximity to NEs	n.s.	0.96	(0.89–1.03)	n.s.	0.93	(0.85–1.02)
Perceived supportiveness of NEs for PA	n.s.	1.01	(0.88–1.16)	n.s.	1.10	(0.91–1.32)
Childhood experiences	n.s.	0.86	(0.73–1.02)	n.s.	0.90	(0.74–1.11)
Gender						
Male	-			-		
Female	n.s.	0.90	(0.75–1.09)	0.003	0.70	(0.55–0.88)
Age (years)	<0.001	1.04	(1.03–1.04)	<0.001	1.04	(1.03–1.05)
Education level (years)						
≤10 (compulsory)	-			-		
11–13 (high school)	n.s.	1.14	(0.78–1.65)	n.s.	0.92	(0.60–1.44)
>13 (higher education)	n.s.	1.10	(0.77–1.58)	n.s.	0.89	(0.59–1.35)
Currently studying	n.s.	0.97	(0.65–1.46)	n.s.	0.74	(0.46–1.20)
Income (NOK) (6 NOK ≈ 1 US$)						
≤399.000	-			-		
400.000–799.000	n.s.	0.89	(0.68–1.16)	n.s.	0.82	(0.59–1.14)
>800.000	n.s.	1.08	(0.81–1.44)	n.s.	1.18	(0.83–1.68)
Missing	n.s.	0.97	(0.67–1.41)	n.s.	0.86	(0.54–1.36)
Co-inhabitation nucleus						
Living with parents/friends	-			-		
Living alone	n.s.	1.07	(0.62–1.84)	n.s.	0.78	(0.36–1.66)
Living with spouse/partner	n.s.	1.27	(0.74–2.16)	n.s.	0.96	(0.45–2.01)
Having small children						
No	-			-		
Yes	n.s.	1.01	(0.81–1.26)	n.s.	0.76	(0.46–1.30)
Region						
Oslo and Akershus	-			-		
Hedmark and Oppland	n.s.	0.82	(0.55–1.23)	n.s.	0.77	(0.46–1.30)
South-eastern Norway	n.s.	0.91	(0.66–1.25)	n.s.	0.94	(0.63–1.39)
Agder and Rogaland	n.s.	1.34	(0.97–1.85)	n.s.	1.17	(0.79–1.73)
Western Norway	n.s.	1.26	(0.95–1.67)	0.014	1.50	(1.07–2.11)
Trøndelag	n.s.	0.99	(0.69–1.41)	n.s.	1.33	(0.86–2.05)
Northern Norway	n.s.	0.84	(0.57–1.25)	n.s.	1.09	(0.67–1.75)
Centrality						
Large city	-			-		
Small city	n.s.	0.91	(0.69–1.19)	n.s.	1.24	(0.90–1.73)
Town/village	n.s.	0.96	(0.74–1.25)	n.s.	1.08	(0.78–1.50)
Countryside	n.s.	0.85	(0.63–1.16)	n.s.	1.25	(0.87–1.79)
Municipality’s NE coverage (km^2^)	n.s.	1.03	(0.93–1.15)	n.s.	0.98	(0.86–1.12)
**Explained variance (Nagelkerke *R*^2^) (%)**	**17**			**17**		

Note: n.s. = non-significant.
